# Medical education during the coronavirus disease 2019 pandemic: an umbrella review

**DOI:** 10.3389/fmed.2024.1358084

**Published:** 2024-07-05

**Authors:** Seyed Aria Nejadghaderi, Zohreh Khoshgoftar, Asra Fazlollahi, Mohammad Javad Nasiri

**Affiliations:** ^1^Department of Medical Education, School of Medical Education and Learning Technology, Shahid Beheshti University of Medical Sciences, Tehran, Iran; ^2^HIV/STI Surveillance Research Center, and WHO Collaborating Center for HIV Surveillance, Institute for Futures Studies in Health, Kerman University of Medical Sciences, Kerman, Iran; ^3^Student Research Committee, Tabriz University of Medical Sciences, Tabriz, Iran; ^4^Research Center for Integrative Medicine in Aging, Aging Research Institute, Tabriz University of Medical Sciences, Tabriz, Iran; ^5^Department of Microbiology, School of Medicine, Shahid Beheshti University of Medical Sciences, Tehran, Iran

**Keywords:** medical education, virtual education, COVID-19, dentistry, nursing, pharmacy, veterinary medicine, umbrella review

## Abstract

**Background:**

The coronavirus disease 2019 (COVID-19) pandemic affected many aspects of lifestyle and medical education during the recent years. We aimed to determine the impacts of COVID-19 pandemic on medical education to provide an overview of systematic reviews on it.

**Methods:**

We searched PubMed, Scopus, Web of Science, Cochrane library, Google Scholar, and medRxiv, with the following keywords: “SARS-CoV-2,” “COVID-19,” “Medical Education,” “E-learning,” “Distance Education,” “Online Learning,” “Virtual Education,” “systematic review,” and “meta-analysis,” up to 15 April 2023. Studies were included if they were systematic reviews assessing the impacts of the COVID-19 pandemic on medical sciences students. We used A MeaSurement Tool to Assess systematic Reviews 2 (AMSTAR-2) checklist for quality assessment.

**Results:**

A total of 28 systematic reviews were included. The eligible reviews included between five and 64 primary studies, ranging from 897 to 139,381 participants. Technology-enhanced learning and simulation-based learning were the most frequently used strategies. Virtual teaching has several drawbacks like technical difficulties, confidentiality problems, lower student involvement, connection problems, and digital fatigue. The overall satisfaction rate for online learning was above 50%. Also, favorable opinions about perception, acceptability, motivation, and engagement were reported. The quality of 27 studies were critically low and one was low.

**Conclusion:**

There were reduced clinical exposure and satisfaction for medical students during the pandemic. Further high-quality systematic reviews are required.

## 1 Introduction

Following the World Health Organization declaration on the coronavirus disease 2019 (COVID-19) as a pandemic, different countries have implemented measures like quarantine and lockdown on cities to control the spread of the virus (1). As a results, it has several mental health consequences like anxiety, depression and post-traumatic stress disorder (2). Moreover, fatigue, headache, and attention disorders were three most common long-term adverse events of COVID-19 (3). Following the initiation of COVID-19 vaccination, other complications like thrombotic events and myocarditis were occurred (4).

Following the closure of educational institutes, over 91% of students have been affected (5). It led to a significant learning deficit in students, especially in regions with low socioeconomic status (6). The pandemic also resulted in economic challenges for universities to find money for their staff, facilities, and research projects (7). Transition from face-to-face to online learning leads to challenges and opportunities for teacher education (8). Regarding the medical education, shifting to online distance education, reduced interpersonal interaction and limited opportunities to practice interviewing (9). Furthermore, the written or clinical examinations have been postponed and a debate between open and close book examinations has been arisen (9). There are several concerns for medical students for career choice, including the impossibility of pursuing desired specialties, the removal of elective courses and core rotations during the pandemic (10). On the other hand, roles of medical students in the frontline of the pandemic can lead to gaining clinical experiences about infected patients, despite an increase in exposure and risk of affecting by COVID-19 (11).

While we acknowledge that the COVID-19 pandemic has receded, its long-term effects on medical education, both positive and negative, are still emerging. The lessons learned from this pandemic are crucial for preparing for future health crises that might necessitate quarantines and isolations. Therefore, the topic remains highly relevant. Several previous systematic reviews have evaluated the effects of COVID-19 pandemic on education, satisfaction and assessments of students of medical sciences. However, their findings are dispersed. To our knowledge, no previous umbrella review has comprehensively examined the impact of the COVID-19 pandemic across various fields of medical education. Although several systematic and scoping reviews have explored specific aspects, there has been no study that synthesizes these findings to provide consolidated recommendations and insights. Furthermore, an umbrella review is essential to evaluate the quality of these systematic reviews. This study aims to fill that gap by offering a comprehensive analysis and assessment of existing literature on the subject. Therefore, we aimed to conduct an umbrella review to evaluate the current evidence regarding the medical education during the COVID-19 pandemic.

## 2 Method

### 2.1 Search strategy

We searched PubMed, Scopus, Web of Science, and Cochrane library up to April 15, 2023. Also, the first 300 results of the medRxiv preprint server and the Google Scholar search engine were searched up to April 28, 2033. No limitations on the search fields, such as language, date or study type was implemented. Backward and forward citation searching of the included studies were conducted. The relevant search terms were a combination of the following keywords: (“SARS-CoV-2” OR “COVID-19″) AND (“Medical Education” OR “E-learning” OR “Distance Education” OR “Online Learning” OR “Virtual Education”) AND (“Systematic Review” OR “Meta-analysis”) (Supplementary Table S1).

### 2.2 Study selection

All of the identified articles were exported to the EndNote software version 8.1. Following duplicate removal, two authors independently screened the title and abstracts of the articles. Then, the same ones reviewed the full-texts of the remaining papers. Any discrepancies between the two groups were resolved by discussion or consultation with a third author. The inclusion criteria were those systematic reviews (with or without meta-analysis) evaluating the impact of COVID-19 pandemic on medical education. The exclusion criteria were as follow: (1) study types other than systematic reviews, such as cross-sectional, case-control, cohort or clinical trials; (2) studies using a systematic approach such as living or rapid systematic reviews; (3) systematic reviews on preclinical or animal studies; (4) studies that investigated medical education before the COVID-19 pandemic; and (5) studies not included medical sciences students.

### 2.3 Data extraction

We used a predesigned table in Microsoft Office Word for data extraction. Two researchers extracted the following information from each included study and performed the quality assessment and disagreements were resolved with discussion: basic information (e.g., first author’s name, year of publication and journals), search date and relevant databases, number of included articles, sample size, study designs of the included articles, quality assessment tools, participants’ age and sex, summary of key findings of each study.

### 2.4 Quality assessment

We used “A Measurement Tool to Assess Systematic Reviews 2 (AMSTAR 2)” checklist for quality assessment of included studies (12). Seven of the 16 items on this checklist—protocol registration, adequate literature search, justification for excluding individual studies, risk of bias from the inclusion of individual studies, appropriateness of the meta-analytical methods, consideration the risk of bias when interpreting the review’s findings, and assessment of the presence and likely impact of publication bias—are regarded as critical domains. The checklist does not produce an overall grade; instead, it offers a total assessment based on flaws found in the crucial areas. There are four qualitative levels of confidence in the review’s findings: “high” for no or one non-critical weakness, “moderate” for more than one non-critical weakness, “low” for one critical weakness with or without non-critical weaknesses, and “critically low” more than one critical weakness with or without non-critical weaknesses for the overall level of confidence.

### 2.5 Data synthesis

Due to the high heterogeneity between studies and since most of the included systematic reviews were only reported qualitative data, meta-analysis was not be performed in this study and the data were reported qualitatively and in the form of tables.

## 3 Results

### 3.1 Literature search

**Figure 1:**
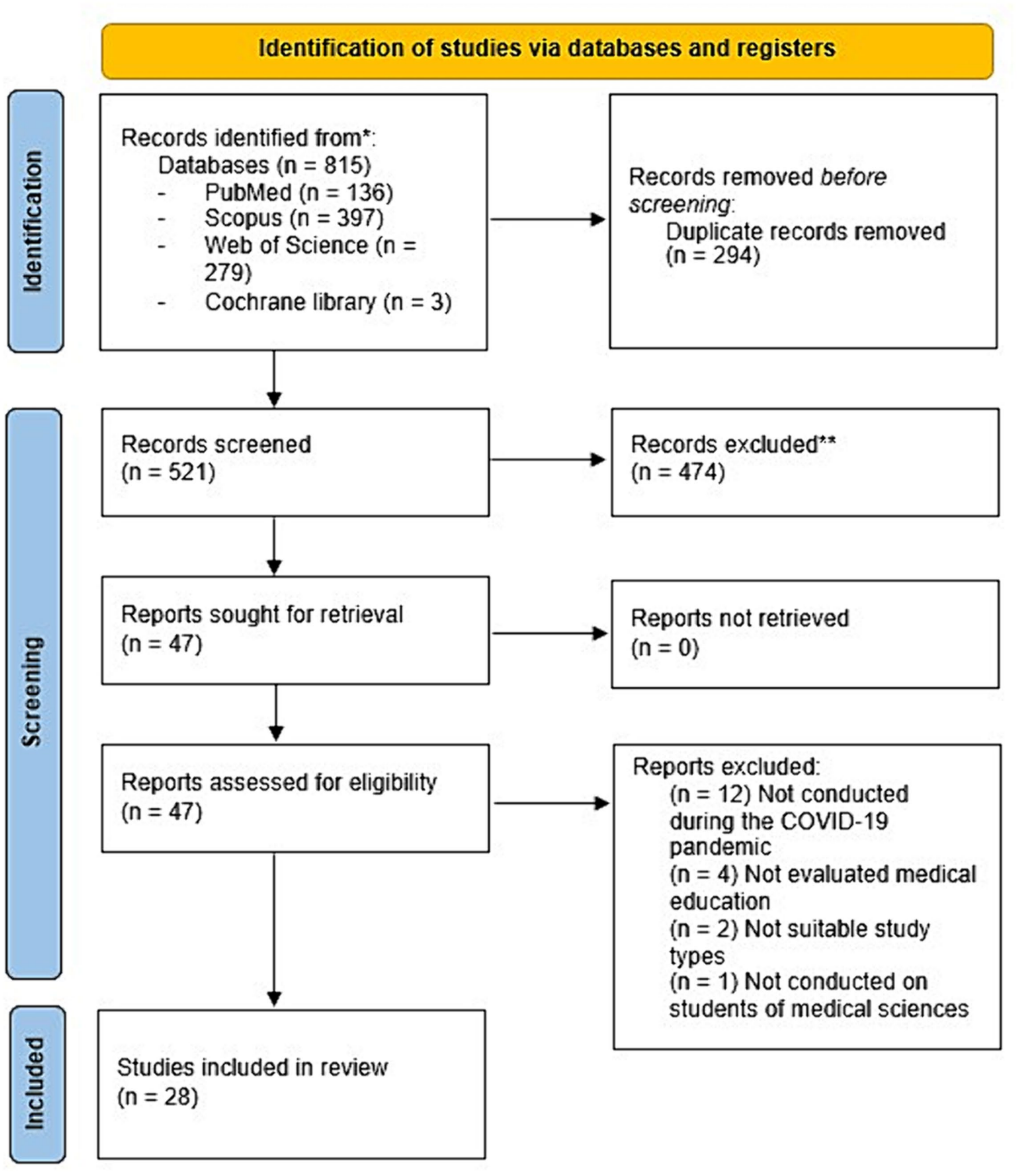
Flowchart of the study selection process. A systematic search yielded 815 studies from PubMed (*n* = 136), Scopus (*n* = 397), Web of Science (*n* = 279), and the Cochrane Library (*n* = 3). After removing duplicates, 521 studies were screened, and 47 were selected for full-text review. Of these, 19 were excluded due to various reasons: 12 were not related to the COVID-19 pandemic, four did not focus on medical education, two had ineligible study designs, and one was not on medical sciences students. Additional hand searching in medRxiv, Google Scholar, and through citation tracking did not yield further studies. In the end, 28 articles met the inclusion criteria.

The systematic search identified a total of 815 studies, which came from PubMed (*n* = 136), Scopus (*n* = 397), the Web of Science (*n* = 279), the Cochrane library (*n* = 3). Following the removal of duplicate studies, the remaining 521 studies were screened and 47 publications were selected for full text review. After evaluating the other 47 articles for eligibility, 19 studies were excluded for the following reasons: 12 studies were not conducted during the COVID-19 pandemic (13–24), four did not evaluate medical education (25–28), two did not have eligible study designs (29, 30), and one was not conducted on students of medical sciences (31). No additional relevant studies were found in medRxiv, Google Scholar, or backward and forward citation searching. Finally, 28 articles met the eligibility criteria and were included (32–59) (Figure 1).

### 3.2 Characteristics of the included studies

The included articles were all published in English and published from 2020 to 2023. They were published in 25 different journals in which BMC Medical Education (*n* = 3) and Medical Teacher (*n* = 2) were the most common ones. The primary studies conducted in almost all continents, most commonly in the United States, the United Kingdom, Canada, and multiple countries. The eligible reviews included between five and 64 primary studies, ranging from 897 to 139,381 participants. Studies used different quality appraisal tools which Newcastle–Ottawa scale (*n* = 2) and Medical Education Research Quality Instrument (*n* = 2) were the most commonly used. Studies evaluated undergraduate, postgraduate or both of them in clinical medicine or its specialties, as well as other health sciences like nursing, pharmacy, veterinary medicine, dentistry, clinical radiography, or mixed of them. Only one study conducted the meta-analysis (41) (Table 1).

**Table 1:** Characteristics of the systematic reviews included in the present umbrella review.

Study identification	Journal	Countries/continents	Search date	Searched databases	Number of included studies/total number of participants	Study design of included studies	Tools for assessment of risk of bias	Age of included participants	Sex of included participants	Major of the participants	Training stage
Dedeilia et al. 2020	*In Vivo*	NR	April 18, 2020	PubMed and EMBASE	61 studies/NR	Case reports, case studies, case control studies, cohort studies, RCTs, letters to the editor, commentaries, editorials, perspectives, “How I do it” reports, reviews or meta-analyses	Critical appraisal of each article between all authors	NR	NR	Medical and surgical education	Undergraduate and postgraduate
Wilcha 2020	JMIR Medical Education	NR	May to June 2020	PubMed and Google Scholar	39 studies/NR	Case reports, case studies, cohort studies, randomized control trials, letters to the editor, commentaries, editorials, and perspectives	NR	NR	NR	Medicine	Undergraduate
Ahmady et al. 2021	Journal of Education and Health Promotion	North America (59%), Europe (21%), Asia (18%), and Oceania (2%)	July 2020	PubMed, Scopus, Web of Science, ERIC	52 studies/NR	Letter to editors, perspectives, experiences, develop guidelines, action plans, mixed methods, and others	QualSyst checklist	NR	NR	Medicine	Undergraduate and postgraduate
Chen et al. 2021	BMC Medical Education	USA, UK, Italy, India, Saudi Arabia, Nigeria, Pakistan, Peru, Canada, Germany, Taiwan, South Korea, and Multiple countries	November 30, 2020	Medline and EMBASE	53 studies/NR	Original articles	NR	NR	NR	Surgical (67.9%), medical (11.3%), interventional (17.0%), multiple specialties (3.8%)	Postgraduate
Hope et al. 2021	Techniques in coloproctology	USA, Italy, Pakistan, India, UK, Chile, France, and Multiple countries	From January 2020 up to August 31, 2020	Medline, EMBASE, PubMed and the Cochrane CENTRAL	29 studies/5,260 trainees and 339 program directors	Original articles	NOS	NR	NR	Surgical specialties except for obstetrics and gynecology	Postgraduate
Islam et al. 2021	Journal of University Teaching and Learning Practice	USA, Malaysia, Nigeria, UK, Libya, Hong Long, India, Germany, Singapore, Indonesia, and multiple countries	June 8, 2020	Google Scholar, PubMed, PubMed Central, and ScienceDirect	17 studies/NR	Opinions, systematic reviews, cross-sectionals, correspondence and communications, round table discussion, surveys, reviews	NR	NR	NR	Veterinary medicine	NR
Lee et al. 2021	European Review for Medical and Pharmacological Sciences	USA, Canada, UK, Italy, Switzerland, Denmark, France, Singapore, Iran, China, Hong Kong, Australia and New Zealand, Brazil, Egypt, and Cameron	June 8, 2020	PubMed, Embase, and ERIC	49 studies/NR	Commentaries, letters, editorials, reviews, research, correspondences, descriptions	NR	NR	NR	Anatomy, Genetics, Surgery, Neurosurgery, Orthopedics, Dermatology, Ophthalmology, health system science	Undergraduate
Naciri et al. 2021	Journal of Educational Evaluation for Health Professions	Pakistan, South Africa, Sri Lanka, UK, India, Saudi Arabia, USA, Turkey, Croatia, Iraq, and China	February 11, 2021	PubMed, ERIC, Science Direct, Scopus, and Web of Science	15 studies/111,622 students	Cross-sectionals	MERSQI	NR	The ratio of male to all participants ranged from 0.30 to 0.71	Medicine, dentistry, and nursing	Undergraduate (46.7%) and postgraduate
Najminouri 2021	Journal of Oral Health and Oral Epidemiology	Iraq, Indonesia, USA, Egypt, Cyprus, China, Singapore, and multiple countries (in North America)	NR	PubMed, Web of Science, Scopus, EMBASE, and Google Scholar	12 studies/NR	Cross-sectionals using questionnaires and interviews	NR	NR	NR	Dentistry	Undergraduate and postgraduate
Nakhoda et al. 2021	Iran Journal of Public Health	Saudi Arabia, Pakistan, Nepal, Qatar, Jordan, China, Canada, Indonesia, Iraq, Croatia, Turkey, Romania, Lebanon, South Korea, Morocco, Mexico, and USA	From 22 December 2019 to 4 January 2021	PubMed, Scopus, Elsevier, Google Scholar, Web of Science, Iranian Scientific Information Database, health.barakatkns, IranDoc, Civilica, and MagIran	24 studies/113,761 participants (7,248 medical and 106,513 non-medical students)	Cross-sectionals	NOS	Mean age ranged from 20.95 to 25.7	Males ranged from 14.9 to 63.3%	Medicine, dentistry, nursing, pharmacy, physiotherapy, health care students, and non-medical students	Undergraduate and postgraduate
Negahi et al. 2021	Clinical Schizophrenia and Related Psychoses	USA, Italy, India, Chile, Pakistan, France, and multiple countries	From December 2000 to May 2021	PubMed, Web of Science, Science Direct, Scopus, and Google Scholar	30 studies/6,776 residents and 220 program managers	Original articles	NR	NR	NR	Surgical specialties	Postgraduate
Santos et al. 2021	Journal of Dental Education	Peru, Costa Rica, USA, France, Serbia, Canada, Brazil, Nepal, China, and multiple countries	September 21, 2020	Cochrane, Embase, Lilacs, Livivo, PubMed, Scopus, Web of Science, Google Scholar, ProQuest, and Open Grey	16 studies/NR	Original articles	JBI Critical Appraisal Checklist for Case Reports	NR	NR	Dentistry	Undergraduate and postgraduate
Abdull Mutalib et al. 2022	BMC Medical Education	NR	Between 23 February 2021 to 23 June 2021	Scopus, ScienceDirect and PubMed	64 studies/139,381 students	Cross-sectionals, qualitative studies, mixed-methods studies, cohorts, RCTs, and case-controls	Alberta Heritage Foundation for Medical Research’s checklist	NR	NR	Medicine, health sciences, dentistry, nursing, veterinary medicine, pharmacy, and multiple majors	Undergraduate
Cartledge et al. 2022	BMC Medical Education	USA, UK, South Africa, Singapore, Iran, Switzerland, Germany, Hong Kong, Canada, Brazil, Spain, UAE, Qatar, Australia, Indonesia, Saudi Arabia, and Bahrain	October 22, 2021	MEDLINE, EMBASE, Google Scholar, and ERIC	36 studies/48 medical schools	Case reports, short reports, and in-practice reports	Author-adapted tool evaluating underpinning bias, setting bias, resource bias and evaluation bias as high quality, unclear quality, or low quality	NR	NR	Medicine	Undergraduate
Grafton-Clarke et al. 2022	Medical Teacher	North America (72.7%), Europe (10.9%), Asia (9.1%), South America (3.6%), Africa (1.8%), and Australia/Oceania (1.8%)	December 21, 2020	Pubmed, EMBASE, CINAHL and PsychInfo	55 studies/mean number of participants: 53.7 (ranged from 2 to 610)	Original articles (60.0%), brief reports/innovations (27.3%), and correspondence articles (12.7%)	Cochrane risk bias tool for RCTs, ROBINS-I, and author-adapted tool evaluating underpinning bias, resource bias, setting bias, educational bias, and content bias	NR	NR	Medical, surgical, and others (i.e., pathology, radiology, pediatrics, primary care, and inter-professional)	Undergraduate (69.1%), postgraduate (27.3%), mixed (3.6%)
Hao et al. 2022	Nurse Education Today	USA (*n* = 7), China (*n* = 3), UK (*n* = 1), Japan (*n* = 1), Korea (*n* = 1), Italy (*n* = 1), Arabia (*n* = 1), and Israel (*n* = 1)	April 2021	PubMed, EMBASE, MEDLINE (OVID), CINAHL and the Cochrane Library	16 studies/1,174 participants (457 nursing and 717 medical students)	Cross-sectionals, quantitative descriptive studies, quasiexperimentals, prospective cohorts, and mixed-method studies	Mixed Methods Appraisal Tool	NR	NR	Medicine and nursing	Undergraduate and mixed
Hsu et al. 2022	Journal of Clinical Medicine	UK (*n* = 12), USA (*n* = 8), Italy (*n* = 5), India (*n* = 5), Germany (*n* = 2), Hong Kong (*n* = 2), Taiwan (*n* = 2), France (*n* = 1), Portugal (*n* = 1), Spain (*n* = 1), Greece (*n* = 1), Switzerland (*n* = 1), Canada (*n* = 1), Ireland (*n* = 1), China (*n* = 1), Malaysia (*n* = 1), Singapore (*n* = 1), and South Korea (*n* = 1)	From 1 January 2020 to 1 October 2021	PubMed	57 studies/NR	Original articles	NR	NR	NR	Orthopedics	Undergraduate and postgraduate
Jain et al. 2022	Neurosurgical Review	96 countries	From December 2019 to December 5, 2020	MEDLINE, PubMed, EMBASE, and Cochrane	26 studies/NR	Original articles (mostly surveys)	Oxford Center for Evidence Based Medicine version 2.1	NR	NR	Neurosurgery	Undergraduate and postgraduate
Lawal et al. 2022	Journal of Medical Imaging and Radiation Sciences	UAE, UK, Nigeria, Ghana, South Africa, Canada, USA, Singapore, Australia, and multiple countries	From July 1 to December 21, 2021	PubMed, Science Direct, CINAHL, and Scopus	17 studies/NR	Cross-sectionals using interviews, focus group discussion, surveys, and questionnaires	QATSDD tool	NR	NR	Clinical radiography	Undergraduate and postgraduate
Loh et al. 2022	JAAD International	UK, USA, Canada, and multiple countries	NR	NR	6 studies/897 participants	Cross-sectionals using surveys	NR	NR	NR	Dermatology	Postgraduate
Papa et al. 2022	Anatomical Sciences Education	UK, Saudi Arabia, Croatia, Israel, Germany, China, Canada, Cyprus, Italy, Malta, USA, Barbados, India, New Zealand, Singapore, South Africa, Turkey, Korea, Brazil, Spain, France, Mexico, Nigeria, Venezuela, Ireland, and multiple countries	Between July 2020 and July 2021	PubMed, Biomed Central, Scopus, and Google Scholar	25 studies/NR	Letters, perspectives, viewpoints, reviews, descriptive articles, monograph, editorials, short communications, insights, reports, and original articles	NR	NR	NR	Medicine and anatomy	Undergraduate and postgraduate
Pires 2022	Pharmacy	Saudi Arabia, Australia, Jordan, China, USA, Canada, UK, UAE, Brazil, Sri Lanka, Spain, Sultanate of Oman, Malaysia, Estonia, and multiple countries	January 2022	PubMed, Cochrane Library, DOAJ, SciELO, and b-on (Online Library of knowledge)	23 studies/about 5,000 participants	Cross-sectional studies using surveys and questionnaires	NR	NR	NR	Pharmacy (and healthcare students)	Undergraduate
Saed 2022	Cureus	USA, Sri Lanka, Hong Kong, and UK	June 2022	PubMed, Medline, and Scopus	18 studies/1,529 participants (ranged from 6 to 763)	Original articles	NR	NR	NR	Medicine (surgical education)	Undergraduate
Shorey et al. 2022	Nurse Education in Practice	USA (*n* = 11), South Korea (*n* = 5), Indonesia (*n* = 4), Jordan (*n* = 4), Iran (*n* = 3), Australia (*n* = 2), Brazil (*n* = 2), Saudi Arabia (*n* = 2), Spain (*n* = 2) and one study each from Canada, China, Croatia, Ireland, Japan, Poland, Malaysia, Singapore, Thailand, South Africa, Turkey, and UK	From December 2019 to September 2022	CINAHL, EMBASE, ERIC, PsycINFO, PubMed and Scopus	47 studies/3,052 students and 241 faculty members	Qualitative and mixed-methods studies	CASP checklist	NR	NR	Nursing	Undergraduate students, faculty members, and both
Stojan et al. 2022	Medical Teacher	USA (*n* = 22), Canada (*n* = 1), Central America (*n* = 1), South America (*n* = 1), Europe (*n* = 5), Asia (*n* = 17), Middle East (*n* = 7), and Oceania (*n* = 2)	December 21, 2020	MEDLINE, EMBASE, CINAHL, and PsychINFO	56 studies/Participants ranged from 6 to 875	Letters to the editor (*n* = 4), brief reports/innovations (*n* = 11), articles/commentaries (*n* = 7), and original research (*n* = 34)	MERSQI	NR	NR	Medicine	Undergraduate, and mixed (undergraduate and postgraduate and undergraduate and faculty members)
Tabatabaeichehr et al. 2022	Journal of Educational Evaluation for Health Professions	Pakistan, Jordan, Indonesia, Morocco, Saudi Arabia, India, South Korea, Nepal, China, Ukraine, Philippines, Greece and Iran	July 10, 2022	Scopus, PubMed, Web of Science, and Persian databases such as Iranmedex and Scientific Information Database	24 studies/15,473 participants	Cross-sectionals	Appraisal tool for cross-sectional studies (AXIS tool)	The percent of males ranged from 14.95 to 100.00%	Mean age range from 19.51 (SD = 1.36) to 22.90 (SD = 2.34)	Medicine, pharmacy, nursing, dentistry, and mixed	NR
Tan et al. 2022	Asia Pacific Scholar	NR	Between 1 February 2020 and 1 September 2020	PubMed	43 studies/NR	NR	NR	NR	NR	Medicine	Undergraduate
Shakeel et al. 2023	Journal of Pakistan Medical Association	USA, Poland, Pakistan, and others	From 2019 to April 2022	Google Scholar, Medline and PubMed	5 studies/NR	NR	NR	NR	NR	Medicine	NR

NR, not reported; RCT, randomized control trial; ERIC, Educational Resources and Information Center; USA, United States of America; UK, United Kingdom; CENTRAL, Central Register of controlled trials; NOS, Newcastle–Ottawa scale; JBI, Joanna Briggs Institute; UAE, United Arab Emirates; CINAHL, cumulated index to nursing and allied health literature; ROBINS, risk of bias in non-randomized studies of interventions; QATSDD, quality assessment tool for studies with diverse designs; DOAJ, directory of open access journals; SciELO, scientific electronic library online; CASP, critical appraisal skills program; MERSQI, Medical Education Research Quality Instrument.

### 3.3 Outcomes

#### 3.3.1 Medicine

Ten studies evaluated the education of undergraduate and postgraduate medical students during this pandemic (32–34, 45, 46, 52, 54, 56, 58, 59). The pandemic had some challenges like decreased motivation and clinical exposure, increased fear, reduction in bed-side teaching and daily ward activities, as well as postponing elective surgeries (32, 58). However, it led to opportunities like use of teleconference, flipped classrooms, virtual consults, live-streaming or recorded surgical procedure videos, development of online resources and peer mentorship, remote clinical visits, multidisciplinary team meetings, and developments of three-dimensional models (32, 33, 46). Technology-enhanced learning and simulation-based learning, as well as small groups and didactics were the most frequently used teaching strategies during the period (34, 56). Several methods for online assessment and clinical examination like simulation programs and video-supervision by clinical educators and comparing the responses with prior studies showed acceptable participants’ responses (32, 45) (Table 2).

**Table 2:** Main findings of the included articles.

	Study identification	General summary
1	Dedeilia et al. 2020	Challenges: 1—There were lack of bedside teaching and students’ direct involvement with patients, which could have optimized physical examination skills and non-technical skill for students, residents, and fellows. 2—There were focus on staffing around emergency medicine, intensive care and general medical specialties. 3—For surgical educations, the elective surgeries were being postponed. 4—Daily activities are drastically reduced in the ward; Implementation of New Technologies: 1—Use of tele-conferences and webinars were promoted. 2—Flipped classrooms and active learning were used, however the transition is difficult for clinical education. 3—Virtual consults, telemedicine, simulation and virtual reality, and social media were used for medical education and patient care. 4—3D models were used for anatomy teaching; Assessments: 1—Assessments were conducted using reinstitution of oral examinations via teleconferences, or through simulation programs, video-supervised by clinical educators
2	Wilcha 2020	The abundance of online resources was one of virtual teaching’s benefits. To enable students to communicate with patients from their homes, new interactive types of virtual teaching are now being created. Students are now able to keep up with the most recent medical developments and recover information that was lost when university courses and clinical attachments were suspended thanks to open-access instruction from medical specialists. With the goals of enhancing knowledge and offering psychological support, peer mentorship has been shown to be an effective technique for medical students. Technical difficulties, problems with confidentiality, lower student involvement, and loss of assessments were drawbacks of virtual teaching
3	Ahmady et al. 2021	The study identified five learning strategies, including TEL, simulation-based learning, technology-based clinical education, mobile learning, and blended learning. It emphasizes that TEL and simulation-based learning were more frequently used than others in distance learning in medical education during the COVID-19 pandemic. These strategies have the potential to increase learners’ level of knowledge and performance by facilitating the use of online learning resources such as Massive Open Online Courses, virtual clinical cases, and blended sources accessible
4	Chen et al. 2021	Decreased clinical experience and reduced case volume; increased working hours and burnout; alterations in educational activities; inadequate personal protective equipment; redeployment to manage the COVID-19 pandemic; failure to meet training requirements; anxiety regarding board exams and career; decreased quality of life and worse mental health
5	Hope et al. 2021	All studies reported decreased operative experience and the redeployment to non-surgical roles ranged from 6.0 to 35.1%; knowledge learning had been moved to online platforms in 17 studies; seven included studies reported trainees had more time to spend on educational/academic activities; and all studies reporting on mental health report negative associations with increased stress, ranging from 54.9 to 91.6% of trainees
6	Islam et al. 2021	Exam cancellations and a quick transition to online learning were among the difficulties faced by veterinary education during COVID-19. It may be conceivable to have online classes for veterinary medical education, however for interactive situational learning of veterinary courses, other factors such as the availability of electronic equipment, student motivation for self-learning, and institutional support are essential
7	Lee et al. 2021	1—Curriculum changes in undergraduate medical education: replacing in-person lectures with online seminars in the preclinical years and using a variety of distance learning strategies to compensate for the reduced duration or cancelled clinical clerkship. 2—Student-led educational activities related to COVID-19: volunteer teams; COVID-19 Medical Student Response Team; Create online initiatives; Student-led peer-mentoring program; Produce a weekly newsletter
8	Naciri et al. 2021	Seven out of 12 studies, which mostly concentrated on technological access, possession of fundamental computer skills, pedagogical design of online courses, online interactions, and learning flexibility, reported generally favorable evaluations. Five of the 12 investigations, however, found primarily unfavorable perceptions, which highlighted barriers relating to internet connections, the use of educational platforms, and the development of clinical abilities. In three out of four investigations, satisfactory levels of acceptance of distant learning were reported. One study found that students’ motivation was comparable to or higher than that of traditional instruction, and another found that students’ involvement increased dramatically during the COVID-19 epidemic. Overall, the results of this study show that students responded favorably in terms of perceptions, acceptability, motivation, and engagement to the emergency switch to online health science learning during this health crisis
9	Najminouri 2021	For dental students, the current study demonstrated that, in order to achieve remote learning, the study environment during the COVID-19 outbreak was mostly dependent on online lessons, teleconferences, and video conferencing. Also, home-based simulation learning (HBSL) and hands-on learning have been the most popular approaches. The students’ overall satisfaction and favorable views about the item “the effect of COVID-19 on theoretical training and knowledge” demonstrated that e-learning had been successful in covering theoretical subjects. It was shown in the section on “the status of clinic training during the pandemic” those alterations to training practical courses caused dental students to be dissatisfied with their performance, have lower self-esteem, and receive insufficient training, so they requested additional and review courses for the training programs
10	Nakhoda et al. 2021	The pooled e-Learning satisfaction in medical, non-medical and overall were 58.1% (50.5–65.7%), 70.1% (66.8–73.5%) and 63.8% (58.9–68.8%), respectively
11	Negahi et al. 2021	Surgical residents’ educational activities, mental health, and surgical activities were all negatively impacted by the COVID-19 pandemic, especially due to a decrease in the operative volume
12	Santos et al. 2021	Dental learners’ attitudes and satisfaction with remote learning as well as learning technology, pedagogical model, and knowledge gain were evaluated. Learning technologies can support continuity in dental education. Poor technical understanding among faculty members, slow Internet connections, and content conversion to online education are among the issues that have been reported
13	Abdull Mutalib et al. 2022	Despite confronting challenges, 50% of the studies’ participants reported being moderately satisfied, 36% extremely satisfied, and 17% not satisfied with their distance learning experience. The majority of research (26%) claimed that online learning was flexible. The most often reported complaints were internet problems (19%) and a lack of connection between students and professors (19%). Students are more engaged in online learning than in traditional one. Two areas were used to evaluate the learning outcome: academic performance and skill development. The majority of research (72%) claimed that online learning boosts academic performance; 14% claimed a decline in performance; 14% claimed there was no impact; and 14% claimed there was an increase in clinical and communication skills. 80% of the studies gathered were rated at level 1 (reaction), 8% at level 2 (learning), and 12% at level 3 (behavior), according to the Kirkpatrick evaluation. Overall, online education outperformed expectations during the pandemic
14	Cartledge et al. 2022	For clinical examinations of medical students, clinical assessments conducted in person (22 studies) or online (14 studies). The use of improved infection control strategies and altered patient participation was described by the authors of research that reported on in-person clinical evaluations. Online software was used to build online examination circuits, according to the authors of research describing online clinical examinations. According to all authors, adapting exams was feasible, results were comparable to student cohorts from prior years, and participant response was favorable. The assessment of the potential for bias revealed variability in the clinical examination reporting
15	Grafton-Clarke et al. 2022	Considering the adaptations in medical education following the COVID-19 pandemic, rapid transitions from workplace-based learning to virtual environments, such as online electives, telesimulation, telehealth, radiology, and pathology image repositories, live-streaming or recorded surgical procedure videos, stepping up of medical students to support clinical services, remote adaptations for clinical visits, multidisciplinary team meetings, and ward rounds, were significant developments. Lack of personal interactions, the absence of standardized telemedicine courses, and the requirement for faculty time, technical resources, and equipment were among the difficulties. Poor reporting of underlying theory, resources, environment, instructional techniques, and content was revealed by the assessment of bias risk
16	Hao et al. 2022	For knowledge and practice, the stand-alone digital education modalities were just as effective as traditional learning. The impact of various instructional technology on medical and nursing interns’ knowledge and practice varies. The quality of the evidence was found to be inconsistent, and the overall risk of bias was high
17	Hsu et al. 2022	For orthopedic education, nearly 90% of students reported that the epidemic had an influence on their academic performance. Redeployment rates of 20.9–23.1% have an impact on training. Emergency or outpatient visits dropped from 18 to 58.6% of total visits. While the rates of elective procedures declined by 43.5–100%, the rates of all surgeries, including emergency surgeries, decreased by 15.6–49.4%. The rate of workload fluctuated between 33 and 66%. Between 50 and 100 percent of surgeons changed their practice. 40.5% of orthopedic doctors reported feeling some light psychological pressure. About 64% had given up on finding research subjects
18	Jain et al. 2022	In addition to the 23 studies that noted the switch to online learning, 8 of them also discussed the redeployment into COVID wards, and 2 of them cited the missing surgical exposure as a result. Three of the seven studies carried out in low- and middle-income countries noted residents’ financial difficulties due to a decline in surgical caseload and the economic downturn. The COVID-19 pandemic has caused a significant interruption in neurosurgery education and training across the globe. Reduced surgical exposure has had a negative effect on educational opportunities. However, developments in virtual technology have made training more accessible and affordable, particularly in low- and middle-income countries
19	Lawal et al. 2022	The papers’ findings were divided into two primary themes: the adoption of new approaches to teaching and learning and the difficulties and resiliency of students during the epidemic. There are some advantages to teaching and learning online, including lower costs and greater flexibility. At the same time, difficulties with platform use, elevated stress levels, and insufficient resources are among the difficulties. The majority of the papers demonstrated that, during the peak of the pandemic, radiography students were eager to support the service delivery initiatives of the clinical departments where they were assigned. However, the students were anxious because the epidemic was unexpected, and they found it difficult to deal with having to wear personal protective equipment all the time while they were at the hospitals. Eight out of 17 studies had high qualities
20	Loh et al. 2022	When dermatology residents engaged in teledermatology consultations and then created an assessment and management plan under the supervision of an attending dermatologist, teledermatology was reported to be helpful for their education
21	Papa et al. 2022	The use of technologies other than cadaveric dissection to teach anatomy was improved by distance learning. Furthermore, there is a distinct divide between those who support dissection and those who think it can be easily overcome or at least incorporated by virtual reality and online learning, both from the perspectives of students and professors. The authors are adamant that thorough resource and technique adaption is necessary for the optimum anatomy teaching practice. However, they are in favor of cadaveric dissection and wish that this pandemic would not completely replace it
22	Pires 2022	Only approximately half of the pharmacy students in the study had favorable opinions about online learning. Pharmacy students seem to have a favorable view of online OSCE courses, and they seem to be realistic and simple to execute. For teaching digital health skills, such as how to conduct online pharmacy consultations, OSCE courses may be especially pertinent. Overall, future e-learning methodologies and/or online course optimization is required
23	Saed 2022	Regarding undergraduate surgical education for medical students, the advancement of virtual learning to a nearly in-person experience is the result of the integration of real-time picture capturing equipment used to display people or items, such as models of wounds. Additionally, when used properly, communication and engagement platforms enable active conversation. However, there are still several obstacles that may be overcome in the future as technology advances, and these go beyond connectivity problems and the limitation of the senses to only two-dimensional sight and sound
24	Shorey et al. 2022	Regarding nursing curriculum change during the COVID-19 pandemic, three key themes were discovered: (1) “Transition to remote and online education,” which highlighted participants’ experiences as turbulent due to academic veracity challenges, technological, and psychosocial challenges; (2) “Acceptance of the un-traveled road,” where participants highlighted the acceptance of remote and online education through flexibility and convenience, multipotentiality, and fostering a spirit of togetherness; and (3) “Hands-on learning,” which highlighted participants’ experiences with hands-on learning. This review revealed that faculties and nursing students had differing perspectives on remote and online education, which eventually led to a difference in how each group experienced the change
25	Stojan et al. 2022	Fewer (*n* = 15) mentioned unique activities whereas the majority (*n* = 41) noted the quick conversion of current products to online formats. Most of them (*n* = 27) had a mix of synchronous and asynchronous elements. Small groups (*n* = 26) and didactics (*n* = 40) were the most popular teaching strategies. Although learner involvement was frequently dynamic, teachers usually incorporated technology to replace and magnify rather than revolutionize learning. Thematic study uncovered both exceptional practices and particular difficulties with online learning. The supporting theory was at the highest risk of bias and the study designs and reporting were of middling quality. Fewer than half of the research (*n* = 23) evaluated changes in attitudes, knowledge, or abilities, and none evaluated behavioral, organizational, or patient outcomes. The majority of the studies (*n* = 54) evaluated response/satisfaction. Undergraduate medical education educators successfully shifted face-to-face instructional methods online and put new ideas into practice. The use of synchronous and asynchronous forms fostered virtual participation while providing flexible, self-directed learning, even though technology’s potential to alter teaching has not yet been completely realized
26	Tabatabaeichehr et al. 2022	Students studying medical science expressed 51.8% satisfaction with e-learning during the COVID-19 pandemic. The level of study, adaptation of course materials, interactivity, understanding of the content, active participation of the instructor in the discussion, use of multimedia in teaching sessions, adequate time dedicated to the e-learning, stress perception, and convenience had significant relationships with medical students’ satisfaction with e-learning during the COVID-19 pandemic. Out of 24 studies, 21 studies were of high quality, 2 studies were of fair quality, and 1 study was of low quality
27	Tan et al. 2022	Medical students have experienced decreased motivation, increased fear, and missed opportunities for clinical exposure as a result of the COVID-19 pandemic. Using social media, virtual or augmented reality technology, video conferencing, and virtual or augmented reality platforms, traditional teaching and evaluation techniques have moved to online platforms. Although it is unclear how effective these solutions will be in the long term, they have already had positive effects on access, time management, and the development of self-directed learning. The absence of actual clinical experiences and patient interaction continues to be a serious problem. Other important concerns mentioned included technical difficulties and digital fatigue
28	Shakeel et al. 2023	Students in their last year needed practical experience to advance in their careers. As a result, this situation has a number of psychological effects, including the difficulty to concentrate during self-study for final-year exam preparation, which results in a loss of identity and self-confidence, as well as the inability to grow into tomorrow’s competent and experienced doctor

COVID-19, coronavirus disease 2019; OSCE, objective structured clinical examination; TEL, technology-enhanced learning.

Virtual teaching has several drawbacks like technical difficulties, confidentiality problems, lower student involvement, restriction of involved senses to sight and sound, connection problems, and digital fatigue (33, 54, 58). However, it can provide flexible, easy access and self-directed learning and improved time management (56, 58) (Table 2).

For anatomy teaching, there is a discrepancy between those support virtual reality and those in favor of cadaver dissection and they recommend that resource and technical developments are necessary for optimal anatomy teaching (52). Another study evaluated the psychological effects of the pandemic on last-year medical students and mentioned loss of identity and self-confidence as the consequences of virtual learning (59) (Table 2).

##### 3.3.1.1 Surgery

Two studies evaluated the effects of COVID-19 on education of residents of surgical specialties (36, 42). The studies reported decreased operative experiences which led to negative effects on mental health, as well as educational and surgical activities (36, 42). Also, the frequency of stress and redeployment to non-surgical roles increased from 54.9 to 91.6% and 6.0 to 35.1%, respectively (36) (Table 2).

##### 3.3.1.2 Orthopedics

The study by Hsu and colleagues evaluated orthopedics education during the pandemic and showed redeployment of 20.9–23.1% participants, 18.0–58.6% decrease in emergency or outpatient visits, and 15.6–49.4% decrease in all surgeries (48). Also, 40.5% of orthopedic doctors reported psychological pressure and 50–100% changed their practice (48) (Table 2).

##### 3.3.1.3 Neurosurgery

Jain et al. reported financial difficulties and educational opportunities for neurosurgery education due to decreased surgical exposure (49). However, virtual technology developments provided accessible and affordable training (49) (Table 2).

##### 3.3.1.4 Dermatology

The article by Loh and colleagues included six studies consisting of 897 dermatology residents revealed that teledermatology can be helpful for their education under the supervision of an attending dermatologist (51) (Table 2).

#### 3.3.2 Dentistry

Two systematic reviews including 12 and 16 studies evaluated the effects of COVID-19 pandemic on undergraduate and postgraduate dentistry students (40, 43) (Table 1). During the pandemic, home-based simulation learning and hands-on learning were the most frequent approaches which reported appropriate coverage of theoretical concepts, while there were dissatisfactions with their practical trainings (40). Overall, online learning technologies can be used to continue dental education despite some problems like low technical understanding among faculty members and slow Internet connections (43) (Table 2).

#### 3.3.3 Pharmacy

The systematic review by Pires on 23 primary studies showed about half of undergraduate pharmacy students did not have satisfying opinions about online learning, whereas objective structured clinical examination was a suitable option for health skills like how to conduct online pharmacy consultations (53) (Table 2).

#### 3.3.4 Nursing

The systematic review by Shorey et al. revealed three changes in nursing curriculum which were transition to online education, acceptance of remote education, and experiences with hands-on learning (55). Undergraduate nursing students and faculty members had various perspectives about this type of education which led to act in different ways (55). Improving remote learning platforms and augmented virtual stimulation are recommended ways for nursing education in pandemics (55) (Table 2).

#### 3.3.5 Veterinary medicine

The systematic review by Islam and colleagues on 17 eligible studies reported that exam cancellation and rapid transition to online learning as the most prominent problems for veterinary students (37). Although it is feasible for veterinary students to have online classes, there are issues such as low availability of electronic equipment and institutional supports (37) (Table 2).

#### 3.3.6 Clinical radiography

The article by Lawal et al. showed some pros (e.g., lower costs and higher flexibility) and cons (e.g., problems with use of platforms and insufficient resources) for clinical radiography students during the pandemic (50) (Table 2).

#### 3.3.7 Mixed disciplines

Seven articles included participants of mixed majors of health sciences (35, 38, 39, 41, 44, 47, 57). One of the studies conducted a meta-analysis to compare the satisfaction rate of medical and non-medical students regarding e-learning which were 58.1 and 70.1%, respectively (41). Moreover, 36 and 50% of undergraduate health science students were extremely and moderately satisfied with online education, respectively (44). In addition, 72% reported improvements in academic performance and 14% in clinical skills (44). In this regard, online education was as effective as traditional methods in terms of knowledge and practice (47) with a satisfaction rate of 51.8% for medical sciences students (57). Favorable opinions about perception, acceptability, motivation, and engagement have been reported (39), while it had some issues like burnout and decreased quality of life and anxiety for the exams (35, 39). To deal with the transition, some student-led educational activities like COVID-19 medical student response team and student-led peer-mentoring program have been developed (38) (Table 2).

### 3.4 Quality assessment

The quality assessment results showed 27 (96.4%) were critically low and one (3.6%) was low. Among the critical domains, the most common problem was not considering to report the source of funding in the included studies and justifying and discussing the observed heterogeneity in the included studies (Supplementary Table S2).

## 4 Discussion

The results of our umbrella review on 28 systematic reviews showed a reduction in clinical and surgical exposure, as well as bed-side teaching which were the most common problems of online education. There were some problems like technical problems and insufficient resources with virtual learning. The satisfaction of medical students with online learning was low to moderate and clinical skills need the most attention.

In accordance with our findings, the results of an international survey on 1,604 participants from 45 countries showed that 81.4% of participants reported negative impacts of COVID-19 on medical education (60). We also found that reduced educational activities and surgeries, especially for surgery-related specialties led to dissatisfaction, psychological pressure, and redeployment. The abovementioned study also found that reduced in-person and ward teaching had a significant negative impact on medical education (60). Several alternative methods like problem-based learning techniques, virtual meetings, remote clinical visits, or live-streaming procedures were also developed in response to COVID-19 (61, 62). Moreover, results of one of the studies on clinical examination of medical students showed comparable results with before the COVID-19 pandemic (45). Also, postponing or cancellation of exams were other problems caused by the pandemic for students of medical sciences. In this regard, there are debates whether use open or closed book examinations and methods used for grading (9). It seems that both types of open and closed book examinations can be used for blended assessment during or post-pandemics (9).

COVID-19 had several consequences on mental health of students of health sciences like increase of fear and depression. In the same way with our findings, a meta-analysis of 41 studies on 36,608 medical students showed pooled prevalence of 37.9 and 33.7% for depression and anxiety among medical students, respectively (63). Furthermore, it led to higher levels of stress, in addition to emotional and behavioral changes among medical students (64). In an effort to encourage prospering during COVID-19, innovative wellness initiatives and mental health counseling programs for medical students are suggested (65).

Dental students were not satisfied with practical and hands-on practices during the pandemic, as our results suggested. Arponen et al. who evaluated the dentistry students’ performance on examinations showed no significant improvement in examinations of undergraduate dentistry students during the COVID-19 pandemic (66). The differences could be due to study design and number of participants in the studies. To improve the quality of teaching strategies like development of virtual reality, inter-institutional training programs, virtual computerized patients, and facilitating access to online learning resources are recommended (67). Adaptation in the curriculum of dental education with embracing new technologies and simulation-based training should be also considered after the pandemic (68).

Our findings showed dissatisfaction about online learning in approximately half of pharmacy students. Results of a questionnaire on 482 pharmacy students showed limited hospital training, problems with concentration for a long time, and technical problems like the Internet access problems and poor gadgets functioning as barriers of virtual learning during the COVID-19 pandemic (69). Implementation and development of interactive pedagogical methods like computer-based simulation in pharmacy education can help improvements of pharmacy education in the post-pandemic era (70).

Hands-on learning experiences and online learning transitions were among the changes that were occurred in medical and nursing curriculum during the pandemic. Gaur et al. also revealed transition to emergency remote teaching and assessments environments like virtual simulation and artificial intelligence that can be used in the post-COVID era (71). Previous research suggested five challenges for online education transition, including integration of learning tools, technology access, online proficiency of staff and students, academic dishonesty, and confidentiality and security, that should be considered for improving medical education curriculums (72).

The quality of all of the included systematic reviews in the present study were critically low except for one study with low quality. Previous umbrella reviews which were conducted on different aspects of COVID-19 showed that most of the studies had critically low, low, or moderate quality (73–75). Therefore, it sounds that the primary and secondary studies that were conducted during the COVID-19 pandemic had high risk of bias, so further high-quality research are required. Also, it should be considered that the findings should be interpreted with caution.

Despite conduction of several systematic reviews on the effects of COVID-19 pandemic on medical education, to our best of knowledge, no previous umbrella review was conducted to evaluate the quality of them and summarize the findings. So, it is one of the pioneer studies that was conducted on systematic reviews on medical education and COVID-19. However, it has several limitations that should be considered. Firstly, most of our studies were focused on students of medicine, while there are limited ones on dentistry, nursing, pharmacy, and veterinary medicine students. Therefore, further studies on other specialties are suggested. Secondly, despite searching different databases and conduction of grey literature search, we cannot rule out the possibility of missing some suitable studies. Also, we searched for preprints that are not peer-reviewed in order to reduce the possibility of missing relevant systematic review, but no eligible study was found in medRxiv. Thirdly, only one study conducted meta-analysis, so we could not perform meta-analysis and only conducted qualitative synthesis. Fourthly, the protocol of the umbrella review was not registered in the International Prospective Register of Systematic Reviews (PROSPERO) due to the necessity to conduct and report the findings soon. Nevertheless, it was submitted to and approved by the relevant committee in the university. Fifthly, the age and sex of participants included in the systematic reviews were not reported, as a result we could not prepare the COVID-19 impacts on medical education by age and sex. Sixthly, the included studies did not report data on second-tier courses (e.g., legal medicine). So, the specific data on these courses were not provided in the current umbrella review. It is suggested that future original articles and systematic reviews consider evaluation of the effects of the COVID-19 pandemic on these types of courses.

## 5 Conclusion

There were reduced clinical exposure during the pandemic, so teleconference and flipped classrooms were most used for virtual teaching. There was reduced satisfaction for medical students, especially for clinical skills, while online education was effective for knowledge. Further high-quality systematic reviews on the effects of COVID-19 pandemic on medical education are recommended.

## Data availability statement

The original contributions presented in the study are included in the article/Supplementary material, further inquiries can be directed to the corresponding author.

## Author contributions

SN: Conceptualization, Data curation, Formal analysis, Investigation, Methodology, Resources, Software, Validation, Visualization, Writing – original draft, Writing – review & editing. ZK: Conceptualization, Methodology, Project administration, Resources, Supervision, Validation, Writing – original draft, Writing – review & editing. AF: Data curation, Writing – original draft, Writing – review & editing. MN: Conceptualization, Investigation, Resources, Supervision, Validation, Writing – original draft, Writing – review & editing.
