# Supplementary material for: Medical education during the coronavirus disease 2019 pandemic: an umbrella review
Source: Front Med (Lausanne). 2024 Jul 5;11:1358084. doi: 10.3389/fmed.2024.1358084 (PMC11257851; doi:10.3389/fmed.2024.1358084)
Supplement: Supplementary file 1 [file Table_1.DOCX]

**Table S1.** Search strategies for the databases.

| **Database**  **(Search date)** | **Step** | **Search strategy** | **Number of results** |
| --- | --- | --- | --- |
| PubMed  (4.15.2023) | #1 | “SARS-CoV-2”[mh] OR “COVID-19”[mh] OR “SARS-CoV-2”[tiab] OR “2019 Novel Coronavirus”[tiab] OR “COVID-19”[tiab] OR “2019-nCoV”[tiab] OR “Coronavirus Disease 2019”[tiab] OR “Coronavirus Disease-19”[tiab] OR “Severe Acute Respiratory Syndrome Coronavirus 2”[tiab] OR “SARS Coronavirus 2”[tiab] OR “Covid19”[tiab] OR “Wuhan Coronavirus”[tiab] | 346,400 |
|  | #2 | “Education, Medical”[mh] OR “Medical Education”[tiab] OR “Education, Distance”[mh] OR “E-learning”[tiab] OR “Electronic Learning”[tiab] OR “Distance Education”[tiab] OR “Distance Learning”[tiab] OR “Online Learning”[tiab] OR “Online Education”[tiab] OR “Electronic Education”[tiab] OR “Virtual Learning”[tiab] OR “Virtual Education”[tiab] OR “Remote Learning”[tiab] OR “Remote Education”[tiab] OR “Clinical Education”[tiab] | 223,761 |
|  | #3 | “Meta-Analysis”[pt] OR “Meta-Analys*”[tiab] OR "Meta-Analyze"[tiab] OR "Metaanalys*"[tiab] OR “Metaanalyze”[tiab] OR “Network Meta-Analysis”[mh] OR “Systematic Review”[pt] OR “Systematic Review”[tiab] OR “Pooled Analysis”[tiab] | 439,997 |
|  | #4 | 1# AND #2 AND #3 | 136 |
| Scopus  (4.15.2023) | #1 | TITLE-ABS-KEY(“SARS-CoV-2” OR “2019 Novel Coronavirus” OR “COVID-19” OR “2019-nCoV” OR “Coronavirus Disease 2019” OR “Coronavirus Disease-19” OR “Severe Acute Respiratory Syndrome Coronavirus 2” OR “SARS Coronavirus 2” OR “Covid19” OR “Wuhan Coronavirus”) | 495,363 |
|  | #2 | TITLE-ABS-KEY(“Medical Education” OR “E-learning” OR “Electronic Learning” OR “Distance Education” OR “Distance Learning” OR “Online Learning” OR “Online Education” OR “Electronic Education” OR “Virtual Learning” OR “Virtual Education” OR “Remote Learning” OR “Remote Education” OR “Clinical Education”) | 485,923 |
|  | #3 | TITLE-ABS-KEY(“Meta-Analys*” OR "Meta-Analyze" OR "Metaanalys*" OR “Metaanalyze” OR “Systematic Review” OR “Pooled Analysis”) | 650,412 |
|  | #4 | 1# AND #2 AND #3 | 397 |
| Web of Science  (4.15.2023) | #1 | TS=(“SARS-CoV-2” OR “2019 Novel Coronavirus” OR “COVID-19” OR “2019-nCoV” OR “Coronavirus Disease 2019” OR “Coronavirus Disease-19” OR “Severe Acute Respiratory Syndrome Coronavirus 2” OR “SARS Coronavirus 2” OR “Covid19” OR “Wuhan Coronavirus”) | 420,963 |
|  | #2 | TS=(“Medical Education” OR “E-learning” OR “Electronic Learning” OR “Distance Education” OR “Distance Learning” OR “Online Learning” OR “Online Education” OR “Electronic Education” OR “Virtual Learning” OR “Virtual Education” OR “Remote Learning” OR “Remote Education” OR “Clinical Education”) | 143,405 |
|  | #3 | TS=(“Meta-Analys*” OR "Meta-Analyze" OR "Metaanalys*" OR “Metaanalyze” OR “Systematic Review” OR “Pooled Analysis”) | 668,743 |
|  | #4 | 1# AND #2 AND #3 | 279 |
| Cochrane library (Reviews)  (4.15.2023) | #1 | COVID-19 OR SARS-CoV-2 in Title Abstract Keyword | 65 |
|  | #2 | Medical Education OR E-learning OR Electronic Learning OR Distance Education OR Distance Learning OR Online Learning OR Online Education OR Electronic Education OR Virtual Learning OR Virtual Education OR Remote Learning OR Remote Education OR Clinical Education in Title Abstract Keyword | 622 |
|  | #3 | Meta Analysis OR Systematic Review in Title Abstract Keyword | 4,728 |
|  | #4 | 1# AND #2 AND #3 | 3 |
| medRxiv  (4.28.2023) |  | “COVID-19” AND “Medical Education” AND “Systematic Review” | 4,443 |
| Google scholar  (4.28.2023) |  | (“SARS-CoV-2” OR “COVID-19”) AND (“Medical Education” OR “Electronic Learning” OR “Online Education”) AND (“Meta-Analysis” OR “Systematic Review”) | About 19,300 |

**Table S2.** Quality assessment rating of systematic reviews included in the present review using AMSTAR-2.

| Study identification | AMSTAR-2 assessment | | | | | | | | | | | | | | | | AMSTAR-2 final rating | Funding for systematic review |
| --- | --- | --- | --- | --- | --- | --- | --- | --- | --- | --- | --- | --- | --- | --- | --- | --- | --- | --- |
|  | 1 | 2* | 3 | 4* | 5 | 6 | 7* | 8 | 9* | 10 | 11* | 12 | 13* | 14 | 15* | 16 |  |  |
| Dedeilia et al. 2020 | Y | N | Y | PY | Y | Y | N | N | N | N | NA | NA | N | N | NA | Y | Critically low | NR |
| Wilcha 2020 | N | N | Y | PY | N | N | N | N | N | N | NA | NA | N | N | NA | Y | Critically low | NR |
| Ahmady et al. 2021 | Y | N | N | PY | Y | Y | PY | PY | PY | N | NA | NA | N | N | NA | Y | Critically low | N |
| Chen et al. 2021 | Y | N | Y | PY | Y | Y | N | PY | N | N | NA | NA | N | N | NA | Y | Critically low | N |
| Hope et al. 2021 | Y | Y | Y | PY | Y | Y | N | PY | N | N | NA | NA | N | N | NA | Y | Critically low | NR |
| Islam et al. 2021 | Y | N | Y | PY | N | N | N | PY | N | N | NA | NA | N | N | NA | Y | Critically low | N |
| Lee et al. 2021 | Y | N | Y | PY | Y | Y | PY | PY | N | N | NA | NA | N | N | NA | Y | Critically low | NR |
| Naciri et al. 2021 | Y | Y | Y | PY | Y | Y | N | PY | Y | N | NA | NA | N | N | NA | Y | Critically low | N |
| Najminouri 2021 | Y | N | Y | PY | Y | N | PY | PY | N | N | NA | NA | N | N | NA | Y | Critically low | NR |
| Nakhoda et al. 2021 | Y | N | Y | PY | Y | Y | PY | PY | PY | N | N | N | N | N | N | Y | Critically low | NR |
| Negahi et al. 2021 | N | N | N | PY | N | N | PY | PY | N | N | NA | NA | N | N | NA | N | Critically low | NR |
| Santos et al. 2021 | Y | Y | N | PY | Y | Y | Y | PY | Y | N | NA | NA | N | N | NA | Y | Critically low | NR |
| Abdull Mutalib et al. 2022 | Y | N | Y | PY | N | N | PY | PY | Y | N | NA | NA | N | N | NA | Y | Critically low | N |
| Cartledge et al. 2022 | Y | Y | Y | PY | Y | Y | PY | PY | Y | N | NA | NA | Y | N | NA | Y | Critically low | N |
| Grafton-Clarke et al. 2022 | Y | Y | Y | PY | Y | Y | Y | PY | Y | N | NA | NA | Y | N | NA | Y | Critically low | N |
| Hao et al. 2022 | Y | N | Y | PY | N | Y | PY | PY | Y | N | NA | NA | N | N | NA | Y | Critically low | Y |
| Hsu et al. 2022 | Y | N | Y | N | Y | N | N | PY | N | N | NA | NA | N | N | NA | Y | Critically low | Y |
| Jain et al. 2022 | Y | N | Y | PY | Y | N | PY | N | Y | N | NA | NA | N | N | NA | Y | Critically low | NR |
| Lawal et al. 2022 | Y | N | N | PY | Y | N | PY | PY | Y | N | NA | NA | N | N | NA | Y | Critically low | NR |
| Loh et al. 2022 | Y | N | N | N | N | N | N | PY | N | N | NA | NA | N | N | NA | Y | Critically low | N |
| Papa et al. 2022 | Y | N | Y | PY | Y | N | N | PY | N | N | NA | NA | N | N | NA | Y | Critically low | N |
| Pires 2022 | Y | N | Y | PY | N | N | N | PY | N | N | NA | NA | N | N | NA | Y | Critically low | N |
| Saed 2022 | Y | N | Y | PY | N | N | N | PY | N | N | NA | NA | N | N | NA | Y | Critically low | N |
| Shorey et al. 2022 | Y | N | Y | PY | Y | Y | PY | PY | Y | N | NA | NA | N | N | NA | Y | Critically low | N |
| Stojan et al. 2022 | Y | Y | Y | PY | Y | Y | Y | PY | Y | N | NA | NA | Y | N | NA | Y | Low | N |
| Tabatabaeichehr et al. 2022 | Y | N | Y | PY | Y | Y | PY | PY | Y | N | NA | NA | N | N | NA | Y | Critically low | N |
| Tan et al. 2022 | Y | N | Y | N | Y | N | PY | N | N | N | NA | NA | N | N | NA | Y | Critically low | N |
| Shakeel et al. 2023 | N | N | N | N | N | N | PY | PY | N | N | NA | NA | N | N | NA | Y | Critically low | N |

AMSTAR 2 Questions:

1. “Did the research questions and inclusion criteria for the review include the components of PICO?”

2. “Did the report of the review contain an explicit statement that the review methods were established prior to the conduct of the review and did the report justify any significant deviations from the protocol?”;

3. “Did the review authors explain their selection of the study designs for inclusion in the review?”;

4. “Did the review authors use a comprehensive literature search strategy?”

5. “Did the review authors perform study selection in duplicate?”;

6. “Did the review authors perform data extraction in duplicate?”;

7. “Did the review authors provide a list of excluded studies and justify the exclusions?”

8. “Did the review authors describe the included studies in adequate detail?”;

9. “Did the review authors use a satisfactory technique for assessing the risk of bias (RoB) in individual studies that were included in the review?”;

10. “Did the review authors report on the sources of funding for the studies included in the review?

11. “If meta-analysis was performed did the review authors use appropriate methods for statistical combination of results?”

12. “If meta-analysis was performed, did the review authors assess the potential impact of RoB in individual studies on the results of the meta-analysis or other evidence synthesis?”;

13. “Did the review authors account for RoB in individual studies when interpreting/ discussing the results of the review?”;

14. “Did the review authors provide a satisfactory explanation for, and discussion of, any heterogeneity observed in the results of the review?”;

15. “If they performed quantitative synthesis did the review authors carry out an adequate investigation of publication bias (small study bias) and discuss its likely impact on the results of the review?”;

16. “Did the review authors report any potential sources of conflict of interest, including any funding they received for conducting the review?”

*Means critical domains.

AMSTAR-2 final rating

High confidence → No critical or maximum one non-critical weakness // Moderate confidence→ No critical with > 1 non-critical weaknesses

Low confidence→ One critical +/− non-critical weaknesses // Critically low confidence→ > 1 critical +/− non-critical weaknesses

Abbreviations: Y: Yes, N: No, PY: Partial yes; PICO: Population, Intervention, Comparator group, and Outcome; NA: Not applicable (No meta-analysis conducted); NR: Not reported.
